# Suppression of *Listeria monocytogenes* by the Native Micro-Flora in Teewurst Sausage

**DOI:** 10.3390/foods2040478

**Published:** 2013-10-21

**Authors:** Clytrice Austin-Watson, Ar’Quette Grant, Michline Brice

**Affiliations:** 1 Department of Biological Sciences, Delaware State University, Dover, DE 19901, USA; E-Mail: mbrice@umes.edu; 2 Department of Human Ecology, Delaware State University, Dover, DE 19901, USA; E-Mail: arquette.grant@gmail.com

**Keywords:** teewurst, *L. monocytogenes*, micro-flora, PCR-DGGE

## Abstract

Modern consumers are interested in the use of non-chemical methods to control pathogens when heat sterilization is not an option. Such is the case with teewurst sausage, a raw spreadable sausage and a popular German commodity. Although *Listeria* was not found in teewurst, the optimal microbial growing conditions of teewurst coupled with the ubiquity of *L. monocytogenes* in nature, makes the possibility of contamination of products very possible. This pilot study was conducted to examine teewurst’s native micro-flora’s ability to suppress the outgrowth of *L. monocytogenes* at 10 °C using standard plate counts and PCR-DGGE. Traditional plating methods showed *L. monocytogenes* growth significantly decreased when in competition with the teewurst’s native micro-flora (*p* < 0.05). The native micro-flora of the teewurst suppressed the overall growth of *L. monocytogenes* by an average of two logs, under these conditions. Denaturing Gradient Gel Electrophoresis (DGGE) amplicons with unique banding patterns were extracted from DGGE gel for identification. *Brochothrix thermosphacta* and *Lactobacillus curvatus* were identified as a part of the teewurst’s native micro-flora. Although the native micro-flora did not decrease *L. monocytogenes* to below limits of detection, it was enough of a decrease to warrant further investigation.

## 1. Introduction

Food has naturally been a prime medium for microbial growth and proliferation. Although technology and modern safety applications, such as Hazard Analysis Critical Control Points (HACCP), have improved food safety, there is still much to be done as indicated by incidences of food borne illnesses. Modern consumers are interested in more natural, non-chemical, anti-microbial applications. In addition to the use of temperature and packaging, the use of food’s naturally occurring microbes can be used to prevent and/or reduce spoilage organisms and pathogenic growth and proliferation. These applications could result in improved methods of increasing shelf-life. During preparation and storage, the number of some viable bacteria can dwindle while other bacteria continue to grow. For instance, the tight, often vacuumed, packaging of sausages and ready-to-eat meats (RTE) decreases oxygen levels causing a decrease in aerobic organisms and allowing anaerobic bacteria, like lactic acid bacteria (LAB), to thrive [1]*.*

*Listeria monocytogenes*, the causative agent of listeriosis, causes severe problems in pregnant women, neonates, and immunocompromised adults. In the early stages of listeriosis, the symptoms are non-specific flu-like symptoms such as chills, fatigue, headache, muscular/joint pain, and gastroenteritis. Without proper treatment, listeriosis can lead to septicemia, meningitis, encephalitis, spontaneous abortion, and sometimes death [2]. Studies have shown that *Listeria* growth appears to be highly dependent on the temperature and the type and amount of background micro-flora present [3]*.*

The consumption of raw meat and raw meat products are a common occurrence in German culture. Teewurst is a soft-spreadable sausage commodity that is usually served with tea and consumed as a raw, RTE, product. The labels on teewurst indicate if the packaged product is uncooked or cooked, with the former of the two being raw and intended to be cooked by the consumer. The connection between foodborne outbreaks and teewurst has been reported. It was directly cited as a source for illnesses associated with *E. coli* O157:H-resulting in 28 reported cases and three deaths of haemolytic uremic syndrome (HUS) [4]. Teewurst, along with other raw sausages, was recognized as a major risk factor for Shiga toxin producing *E. coli* (STEC) contraction in adults [5]. 

The moisture content (a_w_ ≥ 0.95) and the pH of teewurst sausage (pH ≈ 5.3–5.5) favors microbial growth [6]. Since the native micro-flora is present in teewurst after processing and there are no known outbreaks of *Listeria* associated with this product the behavior of *Listeria monocytogenes* was evaluated to determine if the native micro-flora was robust enough to deter *L. monocytogenes* growth. The optimal microbial growing conditions of the teewurst coupled with the ubiquity of *L. monocytogenes* in nature and its capacity to survive under a wide range of environmental conditions [7], makes the contamination of this product likely.

This was conducted as a pilot study to monitor the viability of *L. monocytogenes* with and without the presence of teewurst’s native micro-flora. The objective of this study was to observe the ability of the native micro-flora to suppress the growth of *L. monocytogenes* in teewurst sausage at abuse temperature.

## 2. Experimental Section

### 2.1. Bacterial Strains

Five strains of *L. monocytogenes* isolates were provided by the Microbial Food Safety Research Unit of the Eastern Regional Research center USDA/ARS, (Wyndmoor, PA, USA); Scott A (serotype 4b, clinical isolate), H7776 (serotype 4b, frankfurter isolate), LM-101M (serotype 4b, beef and pork sausage isolate), F6854 (serotype 1/2a turkey frankfurter isolate), and MFS-2 (serotype 1/2c, environmental isolate from a pork processing plant). The strains were cultured, pooled and maintained as described by Porto, *et al.* [8]*.*

### 2.2. Teewurst Preparation

Teewurst sausage was obtained from a local processor and stored at −20 °C. All teewurst patties were sliced 1.0 cm thick. The patties used to determine the growth of *L. monocytogenes* without competition from the native micro-flora were irradiated by Food Technology Services Inc. (FTSI) (Mullberry, FL, USA) at 25 kGy prior to *Listeria* cocktail inoculation and was labeled as irradiated (I). The non-irradiated (NI) samples did not undergo irradiation and were subjected to the same parameters of the study.

### 2.3. Teewurst Inoculation

Patties were thawed at 4 °C for 24 h. They were then placed onto sterile Styrofoam trays and surface inoculated with 20 μL of *L. monocytogenes* cocktail to simulate surface inoculation that could occur after being sliced with a contaminated blade. The sausage patties were then allowed to air dry at room temperature for 15 min and the inoculation was repeated on the other side. Sausages were packed individually in sterile zipper lock bags and stored at 10 °C. Non-irradiated (NI) control samples were treated under the same conditions as previously explained. On the day of sampling, sausages from control and test samples were weighed and homogenized in 25 mL of sterile 0.1% peptone water (Fisher Scientific, Fair Lawn, NJ, USA) in a lab homogenizer (Telmar STOM 400, Cincinnati, OH, USA) for 1 min. Samples of the control and the test groups were taken on day: 1, 3, 6, 9, 12, and 15, and each set was done in triplicate. 

### 2.4. Microbial Analysis of *Listeria monocytogenes* and Native Micro-Flora

PALCAM medium (Fisher Scientific) supplemented with PALCAM antimicrobial medium base that selected for *Listeria* was used for isolating and cultivating *Listeria* from foods and milk products. Pseudomonas Isolation Agar (PIA) and Lactobacilli MRS agar (Fisher Scientific) was used to detect *Pseudomonas aeruginos*, and other *Pseudomonas* spp., and *Lactobacillus* spp. respectively.

### 2.5. DNA Extraction

DNA was extracted from 1.5 mL of teewurst homogenate. Sample allotments were centrifuged (13,000× *g* for 5 min), the supernatant was removed and the pellet was washed with 1 mL of PBS and centrifuged again. The supernatant was removed and the pellet was re-suspended in 0.5 mL lysis buffer (25% sucrose, 4% 0.5 M EDTA, 5% 1 M Tris-HCl). Samples were incubated for 30 min prior to the addition of 3 μL of 2% proteinase K solution and a 10% SDS was added. After incubation, 0.1 mL of 5 M sodium chloride and 10% (v/v) Cetyltrimithyl-ammonium Bromide (CTab) was added and the mixture was incubated for 30 min at 65 °C. Equal volumes of phenol-chloroform-isoamylalcohol (25:24:1) was added to each tube, shaken vigorously for approximately 30 s and centrifuged for 15 min. The upper aqueous layer was removed and placed in a clean tube. The previous step was repeated using equal volumes of chloroform-isoamylalcohol (24:1). The upper aqueous layer was removed and mixed with 0.8 mL of ice cold isopropanol and centrifugation for 5 min. The supernatant was removed and the remaining pellet was washed in 70% ethanol and centrifuged. The ethanol was carefully removed and samples were allowed to dry. The pellet was re-suspended in TE buffer. DNA of *L. monocytogenes* cocktail cultured in BHI was obtained using the aforementioned method. Samples were stored at −70 °C. 

### 2.6. PCR Amplification

Primers 518r and 357f that amplified the V3 region of the 16S rDNA region were used [9]. A GC-rich clamp was added to the 5′ end of the forward primer resulting in a 250 base pair product. Each reaction consisted of 1× PCR buffer, 1 unit of Taq DNA polymerase, 200 μM of each deopxyribonucleotide trophosphate, 10 ρmole of each primer (Invitrogen, Fredrick, MD, USA), approximately 100 ng template DNA and deionized water to raise the volume to 50 μL. Samples were denatured at 94 °C for 2 min, annealed at 65 °C for 1 min, and extended at 72 °C for 3 min. The annealing temperature was decreased by 1 °C every second cycle until the annealing temperature of 55 °C was reached, upon which an additional 10 cycles were carried out and a final round of extension at 72 °C for 10 min. PCR products were purified as described by Wizard PCR preps DNA purification system (Promega). *L. monocytogenes* isolates were amplified using *L. monocytogenes* specific primers [10] with the PCR method mentioned above.

### 2.7. Denaturing Gradient Gel Electrophoresis (DGGE)

DGGE apparatus and reagents were used as described by DCode Universal Mutation Detection System (Bio-Rad, Hercules, CA, USA). A 40%–50% denaturant range was determined to be the optimum denaturing range for this study. The gel was subjected to electrophoresis of 65 volts for 16 h at 60 °C.

### 2.8. DGGE Amplicon Excision

Amplicons of interest were aseptically excised from the DGGE gel and transferred to 1.5 mL microcentrifuge tubes. A volume of 200 μL of TE buffer was added, and the tubes were shaken for 24 h at 300 rpm. Samples were then cleaned and concentrated as instructed by DNA Clean and Concentrator (Zymo Research, Irvine, CA, USA). These samples were subjected to PCR-DGGE to ensure the same migration patterns. 

The samples were sent to the Delaware Biotechnology Institute (DBI) (University of Delaware, Newark, DE, USA), for pyrosequencing. The sequences were compared in a Basic Local Alignment Search Tool (BLAST). BLAST compared the sequences against the National Institute of Health (NIH) GenBank as part of the International Nucleotide Sequence Database.

### 2.9. Statistical Analysis

Standard plate count data was analyzed using version 9.1.3 of the SAS statistical package (SAS Institute Inc., Cary, NC, USA). Analysis of covariance (ANCOVA) was performed to evaluate the effects of temperature and irradiation on the growth rates of *L. monocytogenes* over time. Mean separations were performed using the Bonferroni LSD method.

NTSYSpc Numerical Taxonomy and Multivariate Analysis System (Stony Brook, NY, USA) was used to perform agglomerate cluster analysis of the DGGE profiles of bacterial communities on a denaturing gradient of 40%–50%. Hierarchical clustering was done by computing a resemblance or similarity (dissimilarity) matrix using the migration distances of the 16S rDNA fragments on the gel. SAHN (sequential agglomerative hierarchical and non-overlapping) was used to compute a resemblance matrix from the data matrix. Statistical analysis used to detect the difference in the microbial shift was done in the same program above. MXCOMP (matrix comparison) was used to compose a Mantel [11] test and to obtain the *p*-value. All *p*-values were set as *p* < 0.05, which indicated a significant difference.

## 3. Results and Discussion

**Figure 1:**
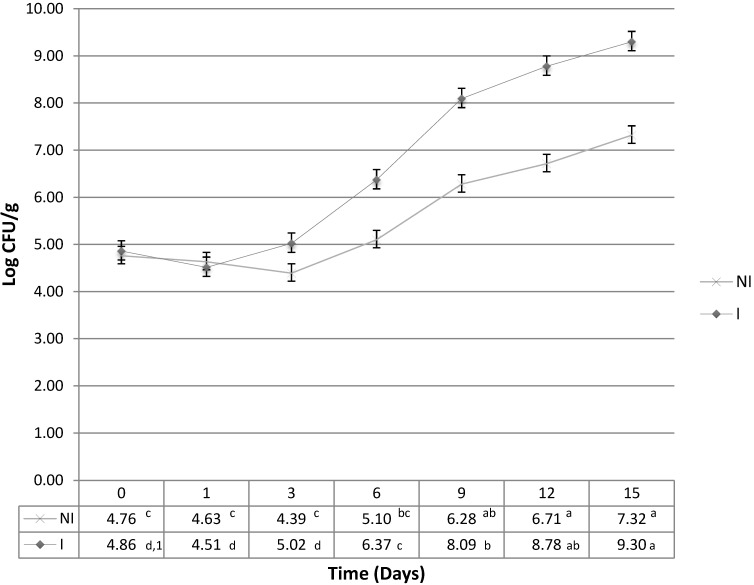
Average growth (Log_10_ CFU/g) of three trials of *L. monocytogenes* in non-irradiated (NI) samples that contain the native micro-flora and irradiated (I) samples, at 10 °C (*n* = 4/sample day). Common letter designation between and amongst groups indicate no significant difference (*p* > 0.05).

This study was able to determine the differences of that suppressed growth, and was also able to show that the difference was significant (*p* < 0.05) between the irradiated and non-irradiated samples (Figure 1). It was expected that *L. monocytogenes* would experience retarded growth in the non-irradiated samples which could possibly be attributed to the growth inhibitions associated with nutrient competition, production of antibacterial compounds, such as lactic acid, and/or other inter-species competitive strategies [12]. It is noteworthy that after day 3, *L. monocytogenes* maintained, on average, a one log increase in the irradiated samples when compared to the non-irradiated samples. Although the growth patterns of *Pseudomonas* spp. was fairly steady and did not exceed maximum population density (MPD) of 6 Log CFU/g, Lactobacillus had a major increase in growth from day 6 to day 9. *Lactobacillus* spp. maintained a steady growth pattern, not exceeding a one log increase between sample days, until day 9 when a 2.5 log increase was observed (results not shown).

Compared to other pathogens, the incidence of reported cases of listeriosis is fairly low, however, the mortality rate of those cases is up to 35% [13]. There is a zero tolerance level of detection in the United States as determined by the USDA Food Safety and Inspection Service (USDA-FSIS) [14]. The elimination of the native micro-flora allowed for an uninhibited growth of *Listeria monocytogenes* under the defined conditions. These results were correlated by Al-Zeyara *et al.* (2010) [15] who showed a significant decrease of *L. monocytogenes* growth when challenged by the native micro-flora in non-selective growth media. *L. monocytogenes* reached a final concentration above 10^3^ cfu/mL when the starting concentration of competing bacteria was less than 10^3^ log cfu/mL. However, when the initial starting concentration of competitors was above 4.5 log cfu/mL, the growth of *L. monocytogenes* was suppressed to undetectable limits when initial concentrations were 10 cells/100 mL.

This study showed that in the absence of background flora, *L. monocytogenes* had a greater MPD than in non-irradiated samples, and maintained a two log difference from sample days nine through fifteen. This increase in *L. monocytogenes* on day 9 between the irradiated and non-irradiated samples coincided with the growth patterns observed with *Lactobacillus* spp. This similarity in growth pattern warrants further research to identify the exact mechanisms of interaction between these organisms. Genetic fingerprints of the background flora were obtained to further identify other organisms, in addition to LAB, that could have participated in the suppression of *L. monocytogenes*. 

**Figure 2:**
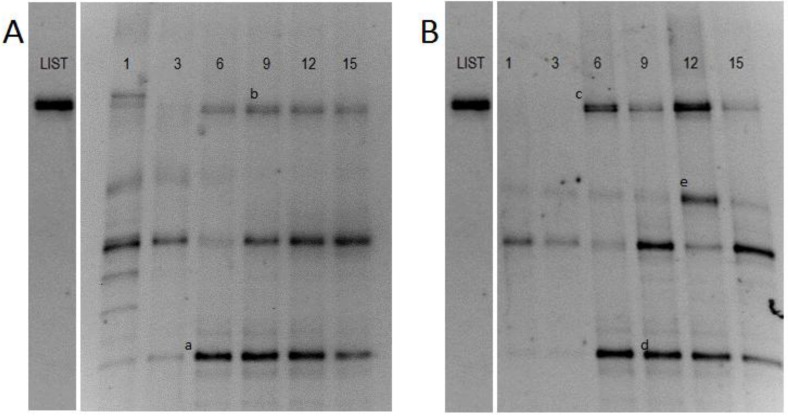
Denaturing Gradient Gel Electrophoresis (DGGE) profiles of PCR product obtained from samples that were not challenged with *L. monocytogenes* (**A**) and samples that were challenged by *L. monocytogenes* (**B**) using a 40%–50% denaturing gradient. *Listeria* markers were used to show the migration pattern of *L. monocytogenes*. Bands indicated by letters were excised and sequenced: (**a**) and (**d**), *B. thermosphacta* (AY543029); (**b**), *Staphylococcus* spp. (HQ711532); (**c**), *Staphylococcus* spp. (HQ71153) and *L. monocytogenes* (FM242711); (**e**), *L. curvatus* (DQ336384).

Teewurst’s genetic fingerpring is illustreated in Figure 2. Pure culture DNA of *L. monocytogenes* was used as a marker to show the migration pattern of *L. monocytogenes* on the DGGE gel. Previous studies determined that *L. monocytogenes* was not naturally occurring in teewurst [6]. Samples that were not inoculated with *L. monocytogenes* saw band migration similar to the *Listeria* marker. After excision and sequencing, amplicon (b) was identified as *Staphylococcus* spp. (HQ711532). PCR using *Listeria* specific primers were also run on the samples not subjected to *L. monocytogenes* cocktail, to determine if *Listeria* was present in samples prior to inoculation. Those results were negative for *Listeria* spp., which correlated to the aforementioned previous study. Amplicon (c) showed a common phenomenon in DGGE referred to as co-migration, where multiple organisms exhibit similar migration patterns [16,17]. Excision and sequencing showed a recovery of both the inoculated *L. monocytogenes* (FM242711) and previously identified *Staphylococcus* spp. (HQ71153). 

Pyrosequencing results indicated LAB was present in teewurst’s background flora (Figure 2). LAB produce bacteriocins that reduce the efficacy of foodborne pathogens by secreting cationic polypeptides and targeting cell membranes thus increase permeability [18,19,20]. Additionally, antimicrobial results could also be caused by the production of acid, nutrient competition, and/or the production of hydrogen peroxide [21]. Zhang *et al.* (2010) [22] illustrated the *Lactobacillus pentosus* produced bacteriocin pentocin 31-1 caused and maintained an average two log cfu/g decrease in growth of *Listeria* over an eighteen day period when compared to the control. Other studies have shown that *L. monocytogenes* is sensitive to bacteriocins and can cause a decrease in efficacy [23], although it would probably not completely eliminate the pathogen. The results of this study support these findings, but more work is necessary to identify bacteriocin production. This assumption was strengthened by the results of this study, as cluster analysis from the DGGE (Figure 2) showed that teewurst’s micro-flora remained, for the most part, constant in the presence of *L. monocytogenes*. Although *L. monocytogenes* grew, the suppression observed may have been due to the presence of LAB presence (results not shown). 

Other organisms identified indicated teewurst’s background flora has bacteria that promote spoilage as well as those that help elongate shelf-life. *B. thermosphacta* is a psychotropic organism that has been cited as one of the primary producers of off-flavors in meat spoilage [24]*.* As a psychrotropic organism, it flourishes at 10 °C. *L. curvatus* is a natural raw meat fermenter that produces lactic acid and is either naturally occurring or supplemented into the meat supply, and it aides in prolonged shelf-life and storage [25]. Although spoilage bacteria were present, such as *B. thermosphacta*, the presence of *L. curvatus* indicated that there may be some microbes present that aide in the storage of teewurst and the suppression of non-native flora. 

## 4. Conclusions

As previously stated, this was a pilot study and more research is needed to identify methods of pathogenic control using teewurst as a model for RTE meats. This study provides insight to potential methods of controlling the growth of pathogens with the presence of native micro-flora. Pyrosequencing methods, similar to Dowd *et al.* (2008) [26], must be conducted to establish a better understanding of the bacteria present in teewurst’s micro-flora. In addition, qRT-PCR can be used to establish a more exact quantification of *L. monocytogenes* and other members of the background flora prior to and after inoculation. 

Promoting the growth of the native flora and/or supplementing bacteriocin producing LAB where thermal processing is not an option could be a route of controlling pathogens in RTE meats. Although teewurst, a specialized ethnic food, was used as a vehicle in this study, the applications can be applied to current food safety concerns in the United States, considering Listeriosis accounts for approximately 1600 cases annually [27]. Such methods have also been applied in common American RTE foods, such as previous work done by Cocolin and Comi [28] to detect *Yersinia* spp. in RTE foods like: raw milk, chocolate milk, carrots, lettuce, tomatoes, and mushrooms. Greater information obtained from this method could help food regulators and producers detect unwanted microbes in their products and show if those unwanted microbes enhance or suppress the native biota present. The information obtained could lead to better understanding of the behavior of listeria, if it were to be introduced to teewurst along the production process. 
